# Immunological characterization of neuronal intranuclear inclusion disease with kidney injury: an exploratory analysis in a multi-center cohort

**DOI:** 10.3389/fimmu.2026.1797076

**Published:** 2026-04-14

**Authors:** Ying Ji, Xiaowen Li, Jin Tian, Xian Chen, Guang Ji, Maofeng Shi, Jing Zhang, Man Xia, Qianru An, Xiang Li, Liangyu Li, Wenjing Song, Ruixue Zhang, Lei Bao, Yuqiao Wang, Yingying Cui, Yuyao Tian, Hao Chen

**Affiliations:** 1 Department of Neurology, The Affiliated Hospital of Xuzhou Medical University, Xuzhou, China; 2 Department of Neurology, Xuzhou Medical University, Xuzhou, China; 3 Department of Neurology, Suzhou Municipal Hospital, Suzhou, China; 4 Nephrology Department, The Second Hospital of Hebei Medical University, Shijiazhuang, China; 5 Department of Neurology, Xuanwu Hospital, Capital Medical University, Beijing, China; 6 Department of Neurology, Yantai Yuhuangding Hospital, Yantai, China; 7 Department of Neurology, Fengxian People’s Hospital, Xuzhou, China; 8 Department of Pathology, The Affiliated Hospital of Xuzhou Medical University, Xuzhou, China; 9 Jiangsu Key Laboratory of Brain Disease and Bioinformation, Research Center for Biochemistry and Molecular Biology, Xuzhou Medical University, Xuzhou, China

**Keywords:** IL-17, IL-6, kidney injury, neuronal intranuclear inclusion disease, neutrophil-to-lymphocyte ratio, NOTCH2NLC, protein misfolding

## Abstract

**Background:**

Neuronal intranuclear inclusion disease (NIID) is a neurodegenerative disorder caused by GGC repeat expansions in NOTCH2NLC, leading to uN2CpolyG protein deposition. Although immune-mediated renal lesions have been described in NIID, the systemic immunoinflammatory profile associated with kidney injury and its relationship with genetic burden remain undefined. This study investigated peripheral immune alterations in NIID-related nephropathy and their correlation with repeat expansion size.

**Methods:**

This multicenter retrospective study enrolled 150 genetically and pathologically confirmed NIID patients from nine tertiary hospitals (2019-2024). Using KDIGO criteria, patients were stratified into NIID with kidney injury (NIID-KD, n=100) and NIID with no kidney injury (NIID-ND, n=50) groups. Peripheral inflammatory markers were assessed in 110 patients, and 12 T-cell-related cytokines were measured in a subset of 35 patients. Multivariable analyses adjusting for disease duration were performed. GGC repeat numbers were quantified in 48 patients.

**Results:**

After adjustment for disease duration, the NIID-KD group maintained significantly higher white blood cell counts, neutrophil counts, monocyte counts, and neutrophil-to-lymphocyte ratio compared to NIID-ND (all *P* < 0.05). Cytokine profiling revealed selectively elevated IL-6 levels in the NIID-KD group (*P* = 0.042), whereas IL-17 elevation did not persist after adjustment (*P* = 0.239). No significant difference in GGC repeat expansion size was observed between groups among genotyped patients.

**Conclusion:**

NIID-associated kidney injury is characterized by a distinct immunoinflammatory signature with sustained neutrophilic and monocytic activation and IL-6 upregulation, suggesting involvement of innate immunity and IL-6-mediated inflammation in renal pathology. The absence of direct correlation between repeat expansion size and kidney involvement indicates that while genetic mutation confers disease susceptibility, acquired inflammatory mechanisms critically determine renal phenotype. These findings provide clinical evidence linking proteinopathy to innate immune activation in NIID.

## 1 Introduction

Neuronal intranuclear inclusion disease (NIID) is a familial neurodegenerative disease and a pathological syndrome of GGC repeat expansions within the 5′ untranslated region of NOTCH2NLC gene (1–3). Its pathological hallmark is the widespread presence of p62/ubiquitin-positive intranuclear inclusions within the central and peripheral nervous systems, as well as in various visceral organs (4). These inclusions contain the misfolded protein uN2CpolyG, which is rich in polyglycine (polyG) (5). With the widespread application of genetic testing and multi-organ biopsies, NIID has been redefined from a “rare central nervous system disorder” to a “systemic intranuclear inclusion disease” affecting multiple organs, including the central and peripheral nervous systems, skeletal muscle, liver, lungs, salivary glands, and the urinary system (6–8).

In recent years, accumulating evidence suggests a close link between misfolded proteins and chronic sterile inflammation (9). Aggregates such as Aβ, α-synuclein, and tau can activate the NLRP3 inflammasome in microglia and peripheral immune cells, triggering the release of pro-inflammatory cytokines like IL-1β and IL-18, thereby driving neuroinflammation and neurodegenerative changes (10–13). uN2CpolyG, as another type of misfolded protein, is also believed to induce chronic inflammatory responses through mechanisms such as overload of protein degradation systems, nucleo-cytoplasmic transport disruption, and DNA damage (14–16). Clinical studies have also observed elevated peripheral inflammatory markers, immunoglobulin abnormalities, and inclusions within immune cells in some NIID patients (17).

The kidney is a classic immune-mediated target organ (18–20). Previous case reports suggest that a minority of NIID patients may present with “lupus nephritis-like” or “crescentic IgA nephropathy-like” glomerular lesions, with some cases showing partial response to glucocorticoids or immunosuppressants (21, 22). Notably, in one reported case, characteristic intranuclear inclusions were identified in a renal biopsy specimen 12 years before the onset of neurological symptoms (7), indicating that the kidney may be one of the early involved organs in NIID. Therefore, NIID-related kidney injury likely represents not merely a “degenerative” or hemodynamic alteration, but is closely associated with an immune-inflammatory process linked to misfolded protein pathology.

Immunologically, the Th17/IL-17 axis has been established to promote glomerular and tubulointerstitial inflammation and fibrosis in inflammatory kidney diseases such as lupus nephritis (23). IL-17 acts on mesangial cells, podocytes, and tubular epithelial cells, inducing the expression of various chemokines and extracellular matrix proteins (23, 24). IL-6, a key cytokine for Th17 differentiation, correlates closely with disease activity in numerous autoimmune nephropathies (23, 25). It remains unclear whether NIID with concomitant kidney injury involves a similar activation of the Th17 immune axis.

Based on a multicenter cohort of NIID patients recruited from nine tertiary hospitals across China, we conducted an immunological sub-study focusing on peripheral inflammatory markers and T cell-related cytokines. This study aimed to compare the immunoinflammatory phenotypes between NIID patients with and without kidney injury and to preliminarily analyze the relationship between NOTCH2NLC GGC repeat expansion burden and renal function parameters. Our objective is to provide clinical insights into the immunological mechanisms underlying renal involvement in NIID.

## 2 Materials and methods

### 2.1 Study subjects and grouping

**Figure 1:**
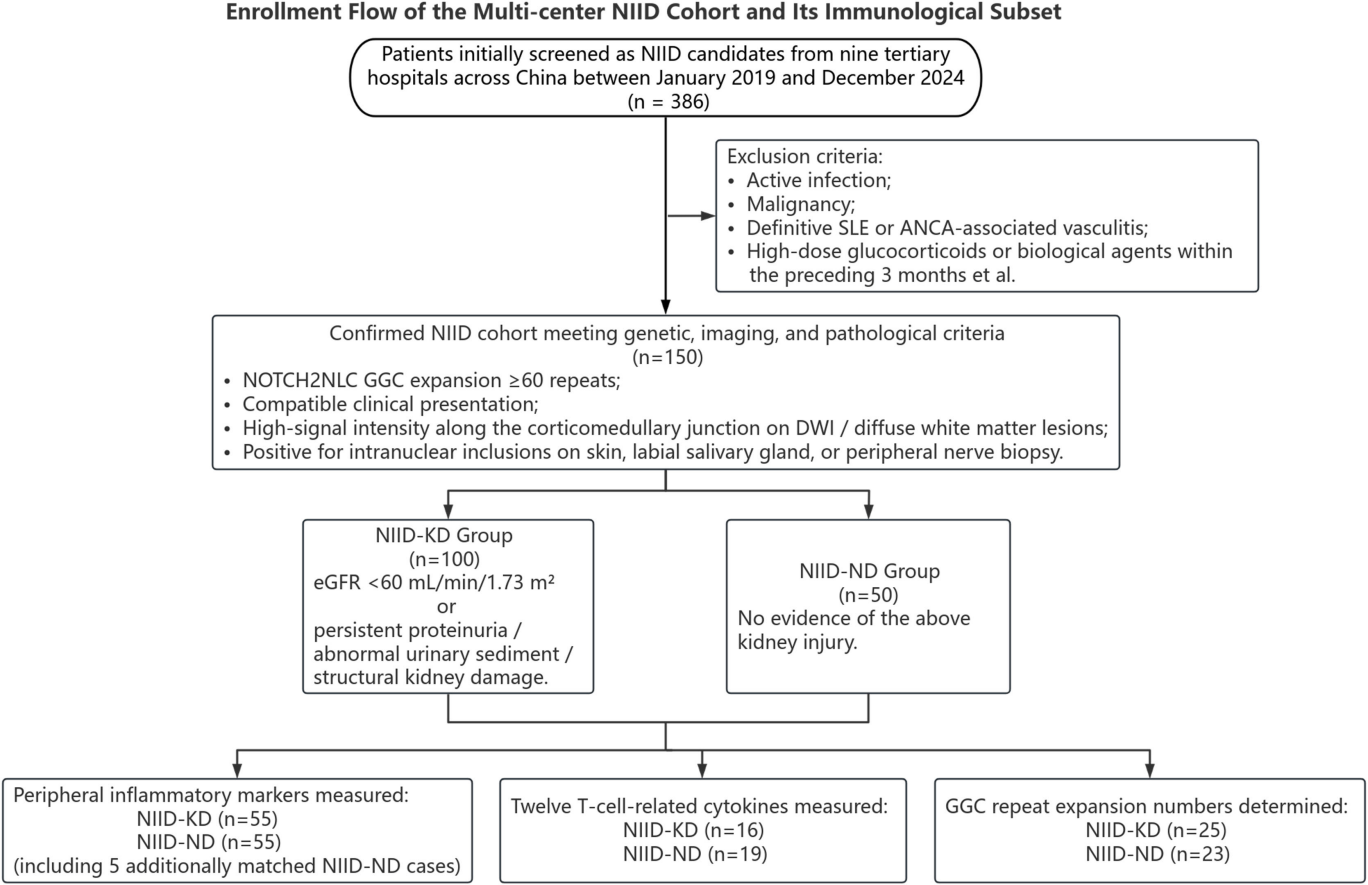
Flowchart of the multicenter NIID cohort and immunological subset enrollment. From January 2019 to December 2024, 386 patients suspected of NIID were identified across nine tertiary hospitals in China. Following screening, 150 patients were confirmed with a diagnosis of NIID based on the presence of NOTCH2NLC GGC repeat expansions, compatible clinical presentations, characteristic brain MRI findings, and positive pathological evidence from at least one biopsy site. According to the KDIGO (Kidney Disease: Improving Global Outcomes) criteria for chronic kidney disease, patients were stratified into a group with kidney injury (NIID-KD, n=100) and a group without kidney injury (NIID-ND, n=50). Among them, 110 patients (NIID-KD, n=55; NIID-ND, n=55) underwent assessment of peripheral inflammatory markers; 35 patients (NIID-KD, n=16; NIID-ND, n=19) were profiled for 12 T-cell-related cytokines; and 48 patients (NIID-KD, n=25; NIID-ND, n=23) had NOTCH2NLC GGC repeat expansion numbers determined. The peripheral inflammatory marker, cytokine, and GGC repeat analysis subsets partially overlap.

This multicenter, retrospective, observational study enrolled 150 adult patients diagnosed with NIID from the neurology departments of nine tertiary hospitals across China between January 2019 and December 2024. To ensure data consistency across centers, all clinical information and laboratory parameters were collected and documented using standardized protocols under equivalent conditions. The cohort enrollment and stratification process are summarized in **Figure 1**. The inclusion criteria were as follows:

1. Genetic confirmation: GGC repeat expansion (≥60 repeats) in the 5′ untranslated region of the NOTCH2NLC gene, verified by repeat-primed polymerase chain reaction (RP-PCR) and GC-rich PCR (26).

2. Clinical presentation: Presence of at least one core clinical phenotype associated with NIID (e.g., cognitive impairment, parkinsonism, cerebellar ataxia, peripheral neuropathy, autonomic dysfunction, stroke-like episodes, or encephalitis-like episodes).

3. Imaging features: Characteristic findings on brain magnetic resonance imaging (MRI), specifically linear high-signal intensity along the corticomedullary junction on diffusion-weighted imaging (DWI) or diffuse white matter lesions (6, 27).

4. Pathological evidence: Identification of p62/ubiquitin-positive and uN2CpolyG-positive intranuclear inclusions in a biopsy specimen from at least one site (skin, labial salivary gland, or peripheral nerve) (4, 28, 29).

Patients were excluded if they had active infections, malignancies, well-defined systemic autoimmune diseases such as systemic lupus erythematosus or ANCA-associated vasculitis, or had received high-dose glucocorticoids or biological agents within three months prior to enrollment.

Based on the KDIGO (Kidney Disease: Improving Global Outcomes) clinical practice guidelines for chronic kidney disease (CKD), patients were categorized into two groups:

1. NIID with kidney injury (NIID-KD) group (n=100): Patients meeting at least one of the following criteria: persistent proteinuria, abnormal urinary sediment, imaging or pathological evidence of structural kidney damage, or an estimated glomerular filtration rate (eGFR) <60 mL·min^-1^·(1.73 m²)^-1^.
2. NIID with no kidney injury (NIID-ND) group (n=50): Patients in whom the aforementioned evidence of kidney injury was systematically ruled out.

To avoid duplication with planned subsequent specialized renal studies, this analysis did not perform further stratification based on glomerular pathology type, CKD stage, or long-term renal outcomes.

### 2.2 Quantification of peripheral inflammatory markers

Fasting venous blood samples were collected from all participants in the absence of significant acute infection or fever. Total white blood cell (WBC) count and differential counts (neutrophils, lymphocytes, monocytes, eosinophils, basophils) were measured using an automated hematology analyzer, and platelet count was recorded. The neutrophil-to-lymphocyte ratio (NLR) was calculated as the absolute neutrophil count divided by the absolute lymphocyte count.

### 2.3 Measurement of T cell-related cytokines

From a subset of consecutively enrolled NIID patients with sufficient stored plasma samples, 35 patients (16 from the NIID-KD group and 19 from the NIID-ND group) were selected for profiling of 12 T cell-related cytokines: IL-1β, IL-2, IL-4, IL-5, IL-6, IL-8, IL-10, IL-12p70, IL-17, IFN-α, IFN-γ, and TNF-α. Measurements were performed using a multiplex flow cytometric bead array or an electrochemiluminescence platform, strictly following the manufacturer’s protocols. All samples were uniformly stored at -80°C and analyzed in a single batch. Laboratory personnel were blinded to the group assignments during the assay.

### 2.4 Analysis of the correlation between GGC repeat expansion number and renal function

Data on NOTCH2NLC GGC repeat expansion numbers were available for 48 patients (25 in the NIID-KD group and 23 in the NIID-ND group). Their renal function parameters, including eGFR, urine specific gravity, and quantitative urinary protein levels, were recorded. Correlation analysis was performed to investigate the relationship between the GGC repeat expansion burden and indicators of renal function impairment.

### 2.5 Statistical analysis

Statistical analyses were performed using SPSS version 26.0 (IBM Corp., Armonk, NY, USA). Normality of continuous variables was assessed using the Shapiro-Wilk test, normally distributed data are presented as mean ± standard deviation and were compared using the independent samples t-test, while non-normally distributed data are presented as median with interquartile range and were compared using the Mann-Whitney U test. Correlations were examined using Pearson’s or Spearman’s coefficient for normally and non-normally distributed data, respectively. To address potential confounding, analysis of covariance (ANCOVA) was employed to adjust between-group comparisons of inflammatory markers and cytokines for baseline characteristics that differed between groups or were considered clinically relevant. For normally distributed variables, ANCOVA was performed on raw values with group (NIID-KD vs. NIID-ND) as the fixed factor and preselected covariates; for non-normally distributed variables, natural logarithmic transformation was applied prior to ANCOVA to approximate normality, with non-parametric alternatives considered if normality was not achieved. Adjusted mean differences (AMD) with 95% confidence intervals (CI) were calculated to quantify the adjusted between-group differences. All tests were two-tailed, and a *P*-value < 0.05 was considered statistically significant.

## 3 Results

The NIID-KD group comprised 43 males and 57 females (male-to-female ratio approximately 3:4), with a mean age of 63.36 ± 6.79 years. The disease duration in this group ranged from 1 to 52 years, with a median of 10 years. The NIID-ND group included 19 males and 31 females (male-to-female ratio approximately 3:5), with a mean age of 61.78 ± 7.70 years. Their disease duration ranged from 0.5 to 31 years, with a median of 6 years. No statistically significant differences were observed between the two groups in terms of sex, age, or the prevalence of underlying conditions such as hypertension, diabetes, and hyperlipidemia (all *P* > 0.05), confirming their comparability (**Table 1**).

**Table 1:** Comparison of baseline characteristics between the NIID-KD and NIID-ND groups.

Variable	NIID-KD group(n=100)	NIID-ND group(n=50)	*P*-value
Demographic parameters			
Male, n(%)	43(43%)	19(38%)	0.558
Age(years)	66.36 ± 6.79	61.78 ± 7.70	0.723
Disease duration(years)	10.00(4.00,14.25)	6.00(4.00,11.50)	0.006**
Comorbidities			
Hypertension, n (%)	54(54%)	24(48%)	0.488
Diabetes mellitus, n(%)	27(27%)	7(14%)	0.073
Hyperlipidemia, n(%)	44(44%)	15(30%)	0.123

Data are presented as mean ± standard deviation, median (interquartile range), or number (percentage) as appropriate. **P < 0.01 indicates statistically significant differences.

### 3.1 Comparison of peripheral inflammatory markers between the two groups

**Figure 2:**
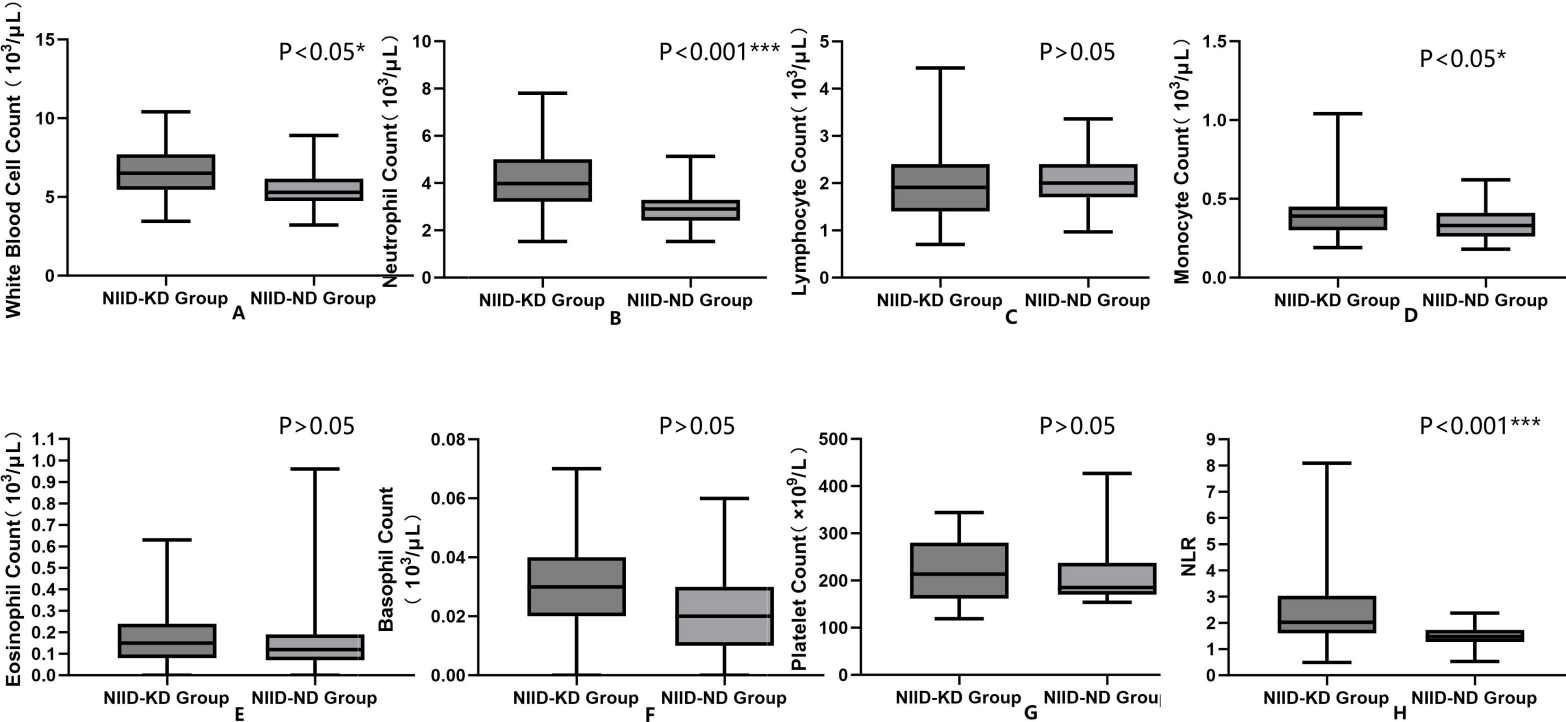
Comparison of peripheral inflammatory markers between NIID-KD and NIID-ND patients. White blood cell count, neutrophil count, and neutrophil-to-lymphocyte ratio (NLR) were significantly higher in the NIID-KD group than in the NIID-ND group **(A, B, H)**. Although monocyte count showed an increasing trend in the NIID-KD group, the difference compared with the NIID-ND group was not pronounced **(D)**. In contrast, lymphocyte count, eosinophil count, basophil count, and platelet count showed no statistically significant differences in distribution between the two groups **(C, E–G)**.

Compared with the NIID-ND group, the NIID-KD group exhibited a more pronounced “neutrophil-dominant” profile in peripheral inflammatory markers (**Table 2**, **Figure 2**): White Blood Cell Count: 5.895 ± 1.254 ×10³/μL in the NIID-KD group vs. 5.467 ± 1.171 ×10³/μL in the NIID-ND group (*P* = 0.027). Neutrophil Count: Significantly higher in the NIID-KD group at 3.335 ± 1.000 ×10³/μL compared to 2.896 ± 0.659 ×10³/μL in the NIID-ND group (*P* < 0.001). Monocyte Count: Slightly higher in the NIID-KD group (0.356 ± 0.141 ×10³/μL) than in the NIID-ND group (0.300 ± 0.073 ×10³/μL) (*P* = 0.047). Neutrophil-to-Lymphocyte Ratio: The NIID-KD group showed a markedly elevated NLR of 1.754 ± 0.626 compared to 1.446 ± 0.284 in the NIID-ND group (*P* < 0.001).

**Table 2:** Comparison of peripheral blood inflammatory markers between the NIID-KD and NIID-ND groups.

Parameter	NIID-KD group(n=55)	NIID-ND group(n=55)	*P*-value
White blood cell count (10^3^/μL)	5.895 ± 1.254	5.467 ± 1.171	0.027*
Neutrophil count(10^3^/μL)	3.335 ± 1.000	2.896 ± 0.659	<0.001***
Lymphocyte count(10^3^/μL)	2.008 ± 0.525	1.945(1.640, 2.250)	0.220
Monocyte count(10^3^/μL)	0.356 ± 0.141	0.300 ± 0.073	0.047*
Eosinophil count(10^3^/μL)	0.169 ± 0.100	0.115(0.070, 0.235)	0.113
Basophil count (10^3^/μL)	0.027 ± 0.015	0.020(0.013, 0.030)	0.079
Platelet count(×10^9^/L)	227.64 ± 71.98	185.00(170.00,237.25)	0.495
NLR	1.754 ± 0.626	1.446 ± 0.284	<0.001***

Data are presented as mean ± standard deviation or median (interquartile range) as appropriate. *P < 0.05, ***P < 0.001 indicate statistically significant differences. NLR, Neutrophil-to-Lymphocyte Ratio.

No statistically significant differences were observed between the two groups for lymphocyte count, eosinophil count, basophil count, or platelet count (all *P*>0.05). This suggests that the observed alterations in the inflammatory profile were predominantly characterized by changes in neutrophil and monocyte counts.

### 3.2 T cell-related cytokine profile: upregulation of IL-6 and IL-17

**Figure 3:**
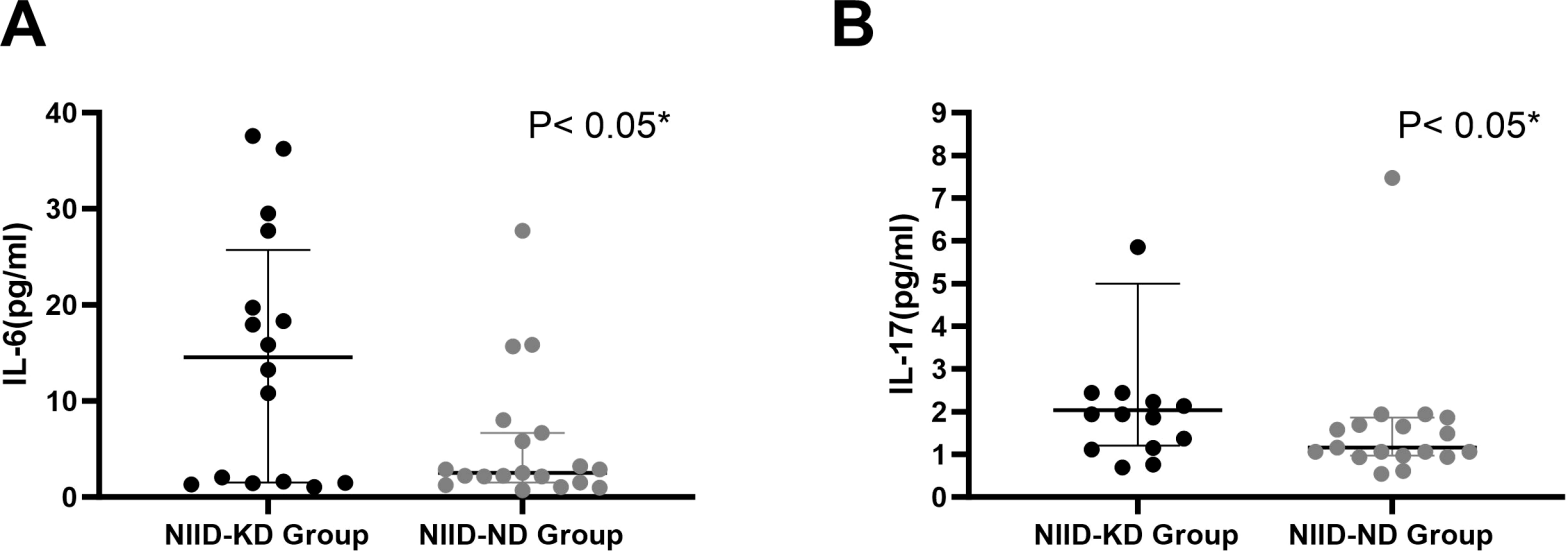
Comparison of IL-6 and IL-17 levels between NIID-KD and NIID-ND patients. Both IL-6 **(A)** and IL-17 **(B)** concentrations were significantly elevated in the NIID-KD group compared to the NIID-ND group.

Among the 35 NIID patients who underwent cytokine profiling, the levels of most T cell-related cytokines were comparable between the two groups, with significant differences observed only for IL-6 and IL-17 (**Table 3**, **Figure 3**): IL-6: Median level was significantly higher in the NIID-KD group at 14.555 (1.523, 25.725) pg/mL compared to 2.280 (1.500, 5.810) pg/mL in the NIID-ND group (*P* = 0.044). IL-17: Median level was significantly higher in the NIID-KD group at 2.035 (1.205, 4.998) pg/mL compared to 1.160 (0.970, 1.860) pg/mL in the NIID-ND group (*P* = 0.026).

**Table 3:** Comparison of 12 T-cell cytokine levels between the NIID-KD and NIID-ND groups.

Cytokine(pg/ml)	NIID-KD group(n=16)	NIID-ND group(n=19)	*P*-value
IL-1β	4.150(2.070, 7.843)	3.480(2.190,5.550)	0.502
IL-2	1.470(0.665, 5.120)	2.020(1.010,20.480)	0.545
IL-4	1.175(0.818, 1.455)	1.050(0.820,2.590)	0.857
IL-5	1.565(1.323, 2.588)	1.700(1.310,2.280)	0.707
IL-6	14.555(1.523, 25.725)	2.280(1.500,5.810)	0.044*
IL-8	4.450(2.468, 23.558)	6.320(2.610,15.770)	0.935
IL-10	0.870(0.695, 1.355)	1.175 ± 0.729	0.883
IL-12p70	1.440(0.885, 2.125)	1.980(1.420,2.230)	0.142
IL-17	2.035(1.205, 4.998)	1.160(0.970,1.860)	0.026*
IFN-α	1.340(0.685, 2.873)	1.410(0.700,2.210)	0.612
IFN-γ	2.275(1.338, 2.950)	3.190(1.680,6.100)	0.182
TNF-α	1.403 ± 0.760	2.398 ± 1.402	0.076

Data are presented as mean ± standard deviation or median (interquartile range) as appropriate. *P < 0.05 indicate statistically significant differences.

No statistically significant differences were found for the other 10 cytokines analyzed (IL-1β, IL-2, IL-4, IL-5, IL-8, IL-10, IL-12p70, IFN-α, IFN-γ, TNF-α; all *P*>0.05). Correlation analysis revealed no stable linear association between IL-6 or IL-17 levels and multiple parameters of renal injury, including eGFR (**Table 4**). This suggests that these cytokines more likely reflect the overall immunoinflammatory activity in NIID patients rather than simply serving as markers of kidney injury severity.

**Table 4:** Correlation analysis between IL-6/IL-17 levels and renal injury indicators in the NIID-KD group.

Indicator parameters	IL-6(pg/ml)		IL-17(pg/ml)	
	*r*	*P*	*R*	*P*
Disease duration (years)	0.111	0.683	0.185	0.493
Serum creatinine (μmol/L)	0.094	0.729	0.231	0.390
Blood urea nitrogen(mmol/L)	0.191	0.478	0.428	0.098
Uric acid(umol/L)	0.174	0.520	0.089	0.742
Cystatin C(mg/L)	-0.182	0.499	0.263	0.325
eGFR(ml/min)	-0.191	0.737	-0.271	0.311
Urine pH	-0.025	0.926	0.085	0.753
Urine specific gravity	0.314	0.236	0.053	0.846
Urine nitrite	-0.168	0.534	-0.126	0.641
Urine leukocyte esterase	0.328	0.214	0.125	0.645
Urine protein	0.332	0.209	0.202	0.454
Urine occult blood	0.319	0.229	0.363	0.167
Urine WBC (counts/μL)	0.277	0.300	0.120	0.657
Casts (counts/μL)	0.150	0.579	-0.090	0.741
Pathologic casts (counts/μL)	0.010	0.972	0.074	0.78

*r*, correlation coefficient; eGFR, estimated glomerular filtration rate; WBC, white blood cells. Correlation analysis was performed using the Spearman (non-normally distributed data) or Pearson (normally distributed data) method. *P < 0.05 indicate statistically significant differences.

### 3.3 Adjusted analyses for disease duration

To address the potential confounding effect of disease duration, which differed significantly between the two groups at baseline (**Table 1**), we performed ANCOVA with group (NIID-KD vs. NIID-ND) as the fixed factor and disease duration as the covariate. After adjustment, the NIID-KD group continued to exhibit significantly higher levels of white blood cell count (AMD = 1.144, 95% CI 0.592-1.697, *P* < 0.001), neutrophil count (AMD = 1.129, 95% CI 0.678-1.580, *P* < 0.001), monocyte count (AMD = 0.064, 95% CI 0.012-0.116, *P* = 0.017), and NLR (AMD = 1.043, 95% CI 0.568-1.517, *P* < 0.001) compared with the NIID-ND group (**Table 5**).

**Table 5:** Adjusted comparison of peripheral inflammatory markers and cytokines between NIID-KD and NIID-ND groups after controlling for disease duration.

Parameter	AMD	95% CI	*P*-value
White blood cell count (10^3^/μL)	1.144	0.592 - 1.697	<0.001***
Neutrophil count(10^3^/μL)	1.129	0.678 - 1.580	<0.001***
Monocyte count(10^3^/μL)	0.064	0.012 - 0.116	0.017*
NLR	1.043	0.568 - 1.517	<0.001***
log IL-6 (pg/ml)	0.844	0.032 - 1.655	0.042*
log IL-17 (pg/ml)	0.376	-0.263 - 1.016	0.239

Data are presented as AMD with 95% CI and *P*-value. Analyses were performed using ANCOVA with group (NIID-KD vs. NIID-ND) as fixed factor and disease duration as covariate. For IL-6 and IL-17, natural log-transformation was applied prior to analysis due to non-normal distribution; the adjusted mean difference represents the difference in log-transformed values. *P < 0.05, ***P < 0.001 indicate statistically significant differences.

For cytokine analyses, because IL-6 and IL-17 were not normally distributed, natural logarithmic transformation was applied prior to ANCOVA. After adjusting for disease duration, log-transformed IL-6 levels remained significantly elevated in the NIID-KD group (AMD = 0.844, 95% CI 0.032-1.655, *P* = 0.042), corresponding to an approximate 2.3-fold increase on the original scale. In contrast, log-transformed IL-17 levels were no longer significantly different between the two groups (AMD = 0.376, 95% CI -0.263-1.016, *P* = 0.239) (**Table 5**). This finding suggests that the elevated IL-17 levels observed in the unadjusted analysis may be partially attributable to the longer disease duration in the NIID-KD group, rather than reflecting a direct association with kidney injury per se. Conversely, the persistent elevation of IL-6 after adjustment indicates a more specific link between this cytokine and renal involvement in NIID, consistent with its established role as a key driver of Th17 differentiation and pro-inflammatory responses.

### 3.4 Preliminary observation of NOTCH2NLC GGC repeat expansions

**Figure 4:**
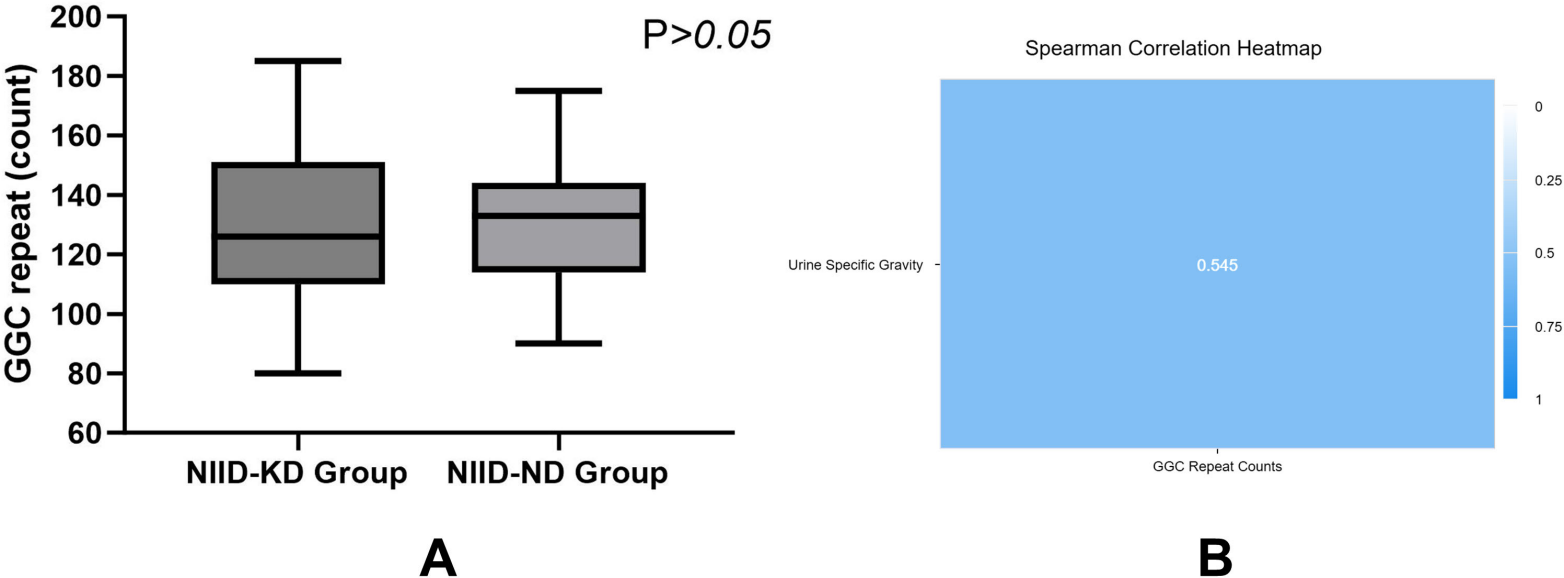
**(A)** Comparison of the frequency distribution of NOTCH2NLC GGC trinucleotide repeat expansion numbers between the NIID-KD and NIID-ND groups showed no statistically significant difference (*P* > 0.05). **(B)** The Spearman correlation heatmap illustrates a significant, moderate positive correlation between GGC repeat expansion number and urine specific gravity in the NIID-KD group (*r* = 0.545, *P* = 0.005).

Among the 48 patients with available GGC repeat expansion data, the overall distribution of repeat expansion lengths was broadly similar between the NIID-KD and NIID-ND groups (**Figure 4A**). No clear linear trend was observed between the GGC repeat expansion number and key renal injury indicators such as eGFR and urinary protein (**Table 6**). A significant positive correlation was observed between GGC repeat expansion number and urine specific gravity (*r* = 0.545, *P* = 0.005; **Table 6**; **Figure 4B**). While this finding may hint at a possible association between repeat burden and renal tubular concentrating function, its biological interpretation remains uncertain. Given the moderate strength of the correlation and the lack of consistent associations with other renal function parameters, this result should be interpreted with caution. Overall, the GGC repeat expansion burden alone appears insufficient to explain the heterogeneity in renal involvement. This finding supports the hypothesis that organ involvement in NIID is shaped by the superposition of acquired immunoinflammatory and comorbidity factors upon a genetic predisposition.

**Table 6:** Correlation analysis between GGC repeat number and renal injury indicators in the NIID-KD group.

Indicator	*r*	*P*-value
Disease duration (years)	0.114	0.587
Serum creatinine(umol/L)	-0.269	0.194
Blood urea nitrogen(mmol/L)	0.058	0.784
Uric acid(umol/L)	0.003	0.989
Cystatin C(mg/L)	-0.253	0.404
eGFR(ml/min)	0.373	0.066
Urine pH	-0.025	0.906
Urine specific gravity	0.545	0.005**
Urine nitrite	-0.044	0.836
Urine leukocyte esterase	-0.193	0.356
Urine protein	0.207	0.3
Urine occult blood	0.221	0.289
Urine WBC (counts/μL)	-0.044	0.836
Casts (counts/μL)	0.098	0.643
Pathologic casts (counts/μL)	0.032	0.877

*r*, correlation coefficient; eGFR, estimated glomerular filtration rate; WBC, white blood cells. Correlation analysis was performed using the Spearman (non-normally distributed data) or Pearson (normally distributed data) method. **P < 0.01 indicates statistically significant differences.

## 4 Discussion

Building upon a multicenter cohort of 150 NIID patients, this study conducted an exploratory analysis of the immunological characteristics in patients with concomitant kidney injury from the perspective of peripheral inflammatory markers and T-cell-related cytokines. The key findings include: First, NIID patients with kidney injury exhibited significant increases in neutrophil and monocyte counts alongside an elevated NLR, suggesting sustained activation of the innate immune system. Second, among 12 T-cell-related cytokines analyzed, only IL-6 and IL-17 were significantly upregulated in the kidney injury group, indicating a selective Th17-axis skewing rather than a global cytokine storm. Third, the burden of NOTCH2NLC GGC repeat expansions was similarly distributed between groups and showed no simple dose-effect relationship with the severity of renal dysfunction, implying that renal involvement is likely a result of superimposed immune-inflammatory and environmental factors acting upon a genetic predisposition.

### 4.1 Immunological evidence for renal involvement in NIID

Previous literature on renal involvement in NIID primarily consists of case reports. Horino et al. described a case of NIID with “lupus-like glomerulonephritis,” where renal biopsy revealed mesangial deposits of IgG, IgA, and C3, resembling lupus nephritis pathomorphologically, yet the patient lacked typical serological features of systemic lupus erythematosus (21). Motoki et al. reported a patient in whom widespread intranuclear inclusions were discovered in a renal biopsy performed 12 years prior to the onset of neurological symptoms (7). Morita et al. documented an NIID case with atypical crescentic IgA nephropathy that responded favorably to corticosteroid therapy (22). Collectively, these cases suggest that renal involvement in NIID is not merely a degenerative change but is closely associated with immune complex deposition and inflammatory responses.

Our cohort-based study supplements these observations. The NIID-KD group demonstrated higher neutrophil and monocyte counts as well as NLR compared to the NIID-ND group, indicating a state of sustained, low-grade systemic inflammation. Concurrently, the selective upregulation of IL-6 and IL-17 points to Th17-axis activation. Integrating these findings with prior pathological evidence of coexisting glomerular IgA/C3 deposits and intranuclear inclusions, a plausible inference can be drawn: the deposition of misfolded uN2CpolyG protein within the kidney may locally activate innate immunity and the Th17 axis, thereby promoting immune complex deposition and glomerular inflammatory responses.

### 4.2 Neutrophils, NLR, and misfolded protein-associated inflammation

The NLR is a simple, stable, and cost-effective peripheral inflammatory marker that has been correlated with disease activity and prognosis in various neurodegenerative and autoimmune disorders (30–33). In NIID, multicenter studies have suggested an association between elevated NLR and cognitive impairment as well as imaging burden (17). Our study further demonstrates that NLR is significantly elevated in NIID patients with kidney injury, accompanied by concurrent increases in neutrophils and monocytes, indicating a persistently activated innate immune state.

Mechanistically, misfolded proteins can act as damage-associated molecular patterns (DAMPs), recognized by pattern recognition receptors to activate the NLRP3 inflammasome, subsequently promoting the release of pro-inflammatory cytokines such as IL-1β and IL-18 (34). Neutrophils not only participate in clearing misfolded proteins and apoptotic cells but can also directly damage the glomerular filtration barrier through the release of neutrophil extracellular traps (NETs) and proteases, amplifying local inflammation (35–40). Therefore, an elevated NLR may serve as a peripheral readout of the misfolded protein-innate immunity axis, which is particularly relevant in NIID given its central pathology of uN2CpolyG aggregation.

### 4.3 The Th17/IL-17 axis: a convergence point between NIID and classical immune-mediated nephropathies

The Th17/IL-17 axis plays a pivotal role in inflammatory kidney diseases such as lupus nephritis (23, 41). IL-17 acts on mesangial cells, podocytes, and tubular epithelial cells, inducing the expression of various chemokines (e.g., CCL2, CXCL1) and fibrosis-related factors (e.g., TGF-β), thereby promoting leukocyte infiltration and extracellular matrix deposition, which drives glomerular and interstitial inflammation and fibrosis (23, 42). Clinical studies also indicate that serum and renal tissue levels of IL-17 correlate with proteinuria and histopathological activity, serving as a potential biomarker for assessing activity in lupus nephritis (41, 43, 44).

In our study, IL-17 and its upstream regulator IL-6 were significantly elevated in NIID patients with kidney injury, while the other 10 cytokines showed no significant change. This pattern suggests that the immune profile of renal involvement in NIID is not a “pan-inflammatory outburst” but rather resembles a selective skewing toward the Th17 axis. This pattern shows some similarity to classical immune-mediated nephropathies. Considering previous reports of “lupus-like” and “IgA nephropathy-like” renal pathology in NIID, it is plausible to speculate that the Th17/IL-17 axis may represent a crucial immunological convergence point between NIID and these nephropathies.

Furthermore, from the perspective of a “misfolded proteinopathy,” we hypothesize that uN2CpolyG aggregation might potentially drive DC/macrophage lineage toward a pro-Th17 polarization via DAMPs. This could, against a predetermined genetic background, transform the originally “static” polyG load into a “dynamic” Th17-mediated renal inflammation. Notably, after adjusting for disease duration, the difference in IL-17 lost statistical significance, suggesting that the longer disease course in patients with kidney injury may partially account for the elevated IL-17 levels observed in unadjusted comparisons. This highlights the importance of considering disease duration when interpreting cytokine profiles in NIID.

### 4.4 Genetic susceptibility and immune shaping: reconsidering the role of GGC repeat expansions

In this study, the distribution of NOTCH2NLC GGC repeat expansion lengths was broadly similar between the NIID-KD and NIID-ND groups. Furthermore, among patients with kidney injury, no clear linear dose-effect relationship was observed between repeat expansion length and renal injury indicators such as eGFR or proteinuria. A more plausible interpretation than “longer expansions lead to worse kidneys” is that the GGC repeat expansion provides the genetic “substrate” for developing uN2CpolyG misfolded proteinopathy. However, the development and severity of kidney injury likely depend more on superimposed acquired immune-inflammatory status and traditional renal/vascular risk factors.

**Figure 5:**
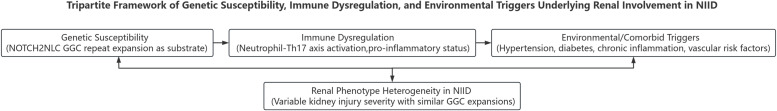
Tripartite framework of genetic susceptibility, immune dysregulation, and environmental triggers underlying renal involvement in NIID. Schematic diagram showing that NOTCH2NLC GGC repeat expansion acts as the genetic substrate; immune dysregulation (neutrophil−Th17 axis activation, pro−inflammatory status) and environmental/comorbid triggers (hypertension, diabetes, chronic inflammation, vascular risk factors) interact dynamically to determine the occurrence and severity of renal injury, thereby explaining the heterogeneous renal phenotypes in NIID patients with similar GGC repeat expansion burdens.

In other words, among individuals with comparable GGC repeat expansion burdens, those who exhibit persistent activation of the neutrophil-Th17 axis and suboptimal control of comorbidities such as hypertension or diabetes are more susceptible to renal involvement. Conversely, the preservation of immunoinflammatory homeostasis and effective comorbidity management may confer protection against renal dysfunction, even in the presence of longer repeat expansions. These observations support our proposed tripartite model, wherein genetic susceptibility, immune dysregulation, and environmental factors collectively contribute to renal phenotype heterogeneity in NIID, offering a potential explanation for the divergent clinical outcomes observed among patients with similar repeat expansion loads (**Figure 5**).

From a clinical management perspective, this implies that for NIID patients, monitoring modifiable immune indicators like NLR, IL-6, and IL-17, and actively managing controllable inflammatory and vascular risk factors, may hold more practical value than merely using GGC repeat numbers to predict renal outcomes. From a mechanistic research standpoint, it suggests future efforts should focus more on the question of “how misfolded proteins are translated into organ-specific injury via immune axes” rather than being confined to the linear thinking of “how long a repeat causes problems.”

Importantly, while a statistically significant correlation between GGC repeat expansion number and urine specific gravity has been observed, this association warrants cautious interpretation. Urine specific gravity is a composite indicator influenced by multiple physiological variables, including hydration status, dietary solute intake, and renal concentrating ability (45). Consequently, it may not directly reflect the pathogenic repeat burden within renal parenchyma. Moreover, the GGC repeat length measured in peripheral blood may not accurately represent the repeat size in kidney cells due to somatic mosaicism, a well-documented phenomenon in repeat expansion disorders (46–49). Tissue-specific variability in repeat length can further obscure genotype-phenotype correlations, particularly in organs such as the kidney where local deposition of uN2CpolyG aggregates and subsequent immune activation may be more pathogenic than the absolute repeat number alone (50). These limitations underscore the necessity for future investigations that incorporate tissue-level genomic and transcriptomic profiling to elucidate the direct impact of repeat expansions on renal pathology.

### 4.5 Study limitations and future perspectives

This study has several limitations. First, limitations inherent to the genetic data and analysis should be considered. While all 150 patients fulfilled the diagnostic criteria for NIID based on pathogenic NOTCH2NLC GGC repeat expansions (≥60), precise repeat-length data were available for only 48 individuals (32%) due to the retrospective multicenter design. This ascertainment bias may affect the genetic subgroup analysis. Among patients with available data, no significant difference in repeat length was observed between the NIID-KD (n=25) and NIID-ND (n=23) groups (*P* > 0.05; **Figure 4A**), suggesting that expansion size does not associate with renal phenotype in this cohort. However, unmeasured systematic differences between patients with and without genetic data cannot be excluded, potentially limiting generalizability. Moreover, limited overlap between patients with repeat-length data and those with inflammatory marker measurements (e.g., NLR, IL-6, IL-17) precluded genotype-immune correlation analyses. Although GGC repeat length correlated with urine specific gravity, this finding should be interpreted cautiously given its unclear biological relevance and the lack of consistent associations with other renal function markers. Somatic mosaicism and tissue-specific repeat variability further complicate interpretation of repeat–phenotype correlations in NIID.

Second, limitations related to the retrospective cross-sectional design and immunoprofiling approach warrant consideration. The cross-sectional design precludes causal inference regarding the relationship between peripheral immune markers and renal disease progression. Cytokine analyses were restricted to a small patient subset without a healthy control group, limiting comparisons to within-cohort observations. Although patients receiving high-dose glucocorticoids or biologics within three months prior to enrollment were excluded, potential immunomodulatory effects of other medications (e.g., ACE inhibitors, ARBs) on inflammatory markers could not be fully excluded. Additionally, systematic renal immunopathological profiling was not performed to avoid overlap with a parallel ongoing study by our team, precluding direct correlation between peripheral immune signatures and the local renal microenvironment.

Third, the absence of standardized neurological phenotyping represents an additional limitation. The relationship between neurological phenotype heterogeneity and renal involvement in NIID was not explored. Such analyses were precluded by the lack of internationally validated consensus on NIID neurological subtyping and the absence of standardized neurological assessments across participating centers in this retrospective cohort. Future prospective studies with harmonized phenotyping protocols are needed to determine whether specific neurological subtypes confer differential risk of kidney injury or exhibit distinct immune profiles.

These limitations should be addressed in future investigations. Prospective longitudinal cohorts with comprehensive genetic characterization and serial immune monitoring integrated with renal function assessment are required to evaluate the prognostic utility of NLR, IL-6, and IL-17 for disease stratification. Integration of multi-tissue genetic analysis and longitudinal renal assessments will be essential to validate repeat-phenotype correlations and elucidate the mechanistic link between repeat expansion and renal dysfunction. Application of single-cell and spatial transcriptomics technologies could further delineate immune cell composition and signaling pathways within NIID renal tissues, validating the pathogenic role of the Th17/IL-17 axis and NETs in local pathology. Finally, mechanistic studies in animal and cellular models are warranted to directly test the impact of the uN2CpolyG-NLRP3-Th17 axis on glomerular and tubular cells, providing an experimental foundation for potential immune-targeted therapies, including inhibitors of IL-17, IL-6, or NLRP3.

## 5 Conclusion

NIID patients with concomitant kidney injury exhibit a distinct systemic immunoinflammatory phenotype, characterized by elevated peripheral neutrophil and monocyte counts, a significantly increased NLR, and selective upregulation of IL-6 and IL-17. The burden of NOTCH2NLC GGC repeat expansions demonstrates a limited correlation with renal function, suggesting that both genetic predisposition and acquired immunoinflammatory factors jointly shape the spectrum of renal involvement. Integrated with previous morphological evidence of “lupus-like” and IgA nephropathy-like renal lesions in NIID, our findings support the hypothesis that a “misfolded protein–innate immunity–Th17 axis” contributes to NIID-related renal pathology. This study provides a foundational basis for subsequent mechanistic investigations and potential immune-targeted therapeutic strategies.

## Data Availability

The raw data supporting the conclusions of this article will be made available by the authors, without undue reservation.
